# Alkane-oxidizing consortia produce substantial amounts of disaccharides

**DOI:** 10.3389/fmicb.2026.1877669

**Published:** 2026-09-09

**Authors:** Stian Torset, Lennart Stock, Marcus Elvert, Manuel Liebeke, Gunter Wegener

**Affiliations:** 1 Max Planck Institute for Marine Microbiology, Bremen, Germany; 2 Alfred Wegener Institute, Helmholtz Center for Polar and Marine Research, Bremerhaven, Germany; 3 Faculty of Geosciences, University of Bremen, Bremen, Germany; 4 Department of Microbiology, Radboud Institute for Biological and Environmental Sciences, Radboud University, Nijmegen, Netherlands; 5 MARUM, Center of Marine Environmental Sciences, University of Bremen, Bremen, Germany; 6 ICBM, Institute for Chemistry and Biology of the Marine Environment, Carl von Ossietzky University Oldenburg, Oldenburg, Germany; 7 Department for Metabolomics, Faculty of Agriculture and Nutrition, University of Kiel, Kiel, Germany

**Keywords:** alkane oxidation, ANME, barrier biome, carbohydrates, carbon cycling, disaccharide, trehalose

## Abstract

Consortia of archaea and partner bacteria couple the anaerobic oxidation of alkanes to sulfate reduction. While the catabolic pathways in anaerobic alkane-oxidizing archaea (ANKA) have been extensively studied, their anabolic capacities remain poorly understood. Here, we examined nine enrichment cultures dominated by ANKA and their partner bacteria for small-molecular compounds using solvent extraction and gas chromatographic analysis of derivatized extracts. All alkane-degrading cultures contained substantial amounts of disaccharides in their metabolite pools. Cold-adapted methane-oxidizing cultures dominated by ANME-2c and Seep-SRB2 contained up to 1.5 mg of trehalose per mg soluble protein. Trehalose was also abundant in ethane-oxidizing cultures of *Candidatus* Ethanoperedens and its distinct partner SRBs, accounting for up to 75% of the extracted metabolites. In contrast, thermophilic ANKA cultures dominated by ANME-1 or *Candidatus* Syntropharchaeum and *Candidatus* Desulfofervidus contained an abundant as-yet-unidentified hexose-containing disaccharide. Metagenomic analysis revealed widespread trehalose metabolism genes among partner *Desulfobacterota* and in ANME-2c and *Ca.* Ethanoperedens, but lower potential in ANME-1 and *Ca.* Syntropharchaeum, consistent with metabolite profiles. When exogenous trehalose was added to a thermophilic ethane-oxidizing enrichment, we observed rapid metabolization by heterotrophic microorganisms but poor assimilation by the *Ca.* Ethanoperedens/*Ca.* Desulfofervidus core community, indicating that ANKA/SRB consortia do not consume externally supplied trehalose. Instead, *Ca.* Ethanoperedens/*Ca.* Desulfofervidus, as well as other ANKA/SRB consortia, may use the disaccharides as energy-storage molecules, osmolytes, or components of the extracellular matrix. The disaccharides produced by the consortia also sustain ancillary heterotrophs, thereby linking alkane oxidation to broader sedimentary carbon cycling.

## 1 Introduction

In deep marine sediments, methanogenic archaea produce substantial amounts of methane (Duan et al., 2023; Reeburgh, 2007). In addition, the thermogenic decay of biomass and abiogenic reactions between hydrogen and carbon dioxide result in the formation of methane and other alkanes (Sherwood Lollar et al., 2002; Tissot and Welte, 1984). These compounds are the energy substrates for anaerobic alkane-oxidizing archaea (ANKA), most of which thrive alongside their sulfate-reducing partner bacteria at the sulfate–methane interface (Boetius et al., 2000; Orphan et al., 2001). Among them, the anaerobic methane-oxidizing archaea (ANME) play a critical role in mitigating methane efflux through anaerobic oxidation of methane (AOM) by consuming up to 90% of the methane in marine sediments (about 380 Tg methane per year; Kurth et al., 2019; Martinez-Cruz et al., 2018; Reeburgh, 2007). ANKA also include archaea that oxidize short- to long-chain alkanes, such as the ethane oxidizers of the *Ca.* Ethanoperedens (Chen et al., 2019; Hahn et al., 2020), and the propane and butane oxidizers of the *Ca.* Syntropharchaeum (Laso-Pérez et al., 2016), with relatively high abundances at select seeps and hydrothermal vents (Wegener et al., 2022).

All ANKA activate their alkane substrate with variants of the methyl-coenzyme M reductase and most are capable of complete substrate oxidation (Wegener et al., 2022). ANMEs achieve this by a complete reversal of methanogenesis, with only minor modifications for the oxidative directionality of the pathway (Hallam et al., 2004; Thauer and Shima, 2008). The enzymatic pathway of non-methane alkane oxidation in archaea is not fully resolved; however, it has been established that due to their lack of a complete respiratory pathway, most ANKA require external electron sinks (Chadwick et al., 2022; Haroon et al., 2013; Knittel and Boetius, 2009; McGlynn et al., 2015). In marine sediments, ANKA form syntrophic consortia with sulfate-reducing bacteria from the phylum *Desulfobacterota* (Boetius et al., 2000; Murali et al., 2023; Schreiber et al., 2010; Wegener et al., 2015). The transfer of reducing equivalents is most likely mediated by direct interspecies electron transfer (DIET; McGlynn et al., 2015; Wegener et al., 2015). However, some alkane-oxidizing archaea do not require syntrophic bacterial partners. For example, freshwater ANME-2d (*Candidatus* Methanoperedens) couples reverse methanogenesis to nitrate or metal oxide reduction (Haroon et al., 2013; McIlroy et al., 2023), whereas *Methanoliparales* couples the degradation of long-chain alkanes to methane formation (Zhou et al., 2021).

Research in the field of anaerobic microbial alkane oxidation revealed the catabolic capabilities of the organisms catalyzing this process, focusing on how the archaeal partners extract energy from alkanes and how they transfer reducing equivalents to their sulfate-reducing partners (Berger et al., 2021; Chadwick et al., 2022; Hahn et al., 2020; Laso-Pérez et al., 2016; McGlynn et al., 2018; McGlynn et al., 2015; Timmers et al., 2017; Zehnle et al., 2023). While small carbon carriers like acetate (McAnulty et al., 2017; Gu et al., 2023) or formate (McAnulty et al., 2017) have been suggested as the source of energy transfer the potential of carbohydrate metabolism of these organisms remains unexplored. This gap is especially apparent after the discovery of ethane-oxidizing archaea, which, under several proposed metabolic schemes, may not generate electron carriers with sufficiently low redox potential to support anabolic CO_2_ fixation (Hahn et al., 2020; Lemaire et al., 2024; Torset, 2025; Wegener et al., 2022). We therefore investigated whether anaerobic alkane-oxidizing communities have the metabolic capacity to produce and metabolize disaccharides, focusing on anabolic pathways and metabolic versatility. Metabolomic analyses revealed a pronounced accumulation of disaccharides across various alkane-oxidizing enrichment cultures, while metagenomics showed that select ANKA and partner bacteria encode numerous pathways for disaccharide metabolism. Stable isotope probing further suggested that ANKA-derived disaccharides may support ancillary heterotrophic community members.

## 2 Methods

### 2.1 Cultivation and maintenance of alkane-oxidizing enrichment cultures

We investigated a collection of anaerobic alkane-oxidizing enrichment cultures. These cultures were produced from sediments sampled at geographically distinct hydrocarbon-rich cold seeps and hydrothermal vents. Such enrichment cultures differ from isolates by the presence of multiple key organisms (i.e., alkane-oxidizing archaea and their partner bacteria) and additionally contain less-abundant ancillary community members. The community composition is described in their respective sources listed in Table 1. These cultures span a diversity of substrates (methane, propane, butane), archaeal lineages, incubation temperatures (12–60 °C), and original depths below sea level (12–1,999 m).

**Table 1:** Description of the here studied enrichment cultures.

Enrichment culture	Dominant ANKA/SRB clade in the consortia	Substrate	Cultivation temperature (°C)	Enrichment pH	Expedition	Year	Location	Coordinates	Water depth (m)	Reference
HR12	ANME-2c/Seep SRB 2	Methane	12	7.2	SO148-1	2000	Cascadia Margin, NE Pacific	44.57 N, 125.145 W	780	Holler et al., 2009
Elba 20	ANME-2c/Seep SRB 2	Methane	20	6.5	Hydra Diving Team	2010	Elba	42.7438 N, 10.118233E	12	Krukenberg et al., 2018
ISIS20	ANME-2a and 2c Seep-SRB1a	Methane	20	7.2	Nautinil Expedition RV L’Atalante	2003	ISIS Mud Volcano	32.350 N 31.383E	1,250	Schreiber et al., 2010
GB37	ANME-1a/Seep SRB2	Methane	37	6.5	AT15-45	2009	Guaymas Basin	27.01 N, 111.409133 W	1999	Krukenberg et al., 2018
GB50	ANME-1a/*Ca.* D. auxilii	Methane	50	6.5	AT15-45	2009	Guaymas Basin	27.01 N, 111.409133 W	1999	Krukenberg et al., 2018
Ethane20	Ethanoperedens unspec./DSS partner	Ethane	20	6.5	AT37-06	2016	Guaymas Basin	27.008 N 111.409 W	2001	Torset, 2025
Ethane50	*Ca.* Ethanoperedens thermophilium	Ethane	50	6.5	AT37-06	2016	Guaymas Basin	27.008 N 111.409 W	2001	Hahn et al., 2020
Butane50	*Ca.* Syntropharchaeum butanivorans/*Ca.* D. auxilii	Butane	50	6.5	AT15-45	2009	Guaymas Basin	27.01 N, 111.409133 W	1999	Laso-Pérez et al. 2018
Propane50	*Ca.* Syntrophoarchaeum caldarius*/Ca.* D. auxilii	Propane	50	6.5	AT15-45	2009	Guaymas Basin	27.01 N, 111.409133 W	1999	Laso-Pérez et al. 2018

The low-temperature methane-oxidizing cultures used here are HR12 and Elba20, both of which were dominated by ANME-2c and Seep-SRB2. HR12 was derived from the Hydrate Ridge, Cascadia Margin, collected at a depth of 780 m during the SO148-1 expedition in 2000 (Holler et al., 2009). Elba20 was established from shallow, methane-percolated sediments near the Italian island Elba at a water depth of 12 m (Hydra Diving Team, 2010; Krukenberg et al., 2018). The ISIS20 culture was established at 20 °C from the ISIS mud volcano (RV L’Atalante, 2003) at 1250 m (ISIS20) and contained ANME-2c, ANME-2a, and ANME-2b with Seep-SRB1a partner bacteria (Schreiber et al., 2010).

All thermophilic cultures were derived from hydrothermally active and hydrocarbon-containing Guaymas Basin sediments. GB50 was established at 50 °C from sediments collected during Atlantis AT15-45 mission in 2009 at a water depth of 1999 m and was dominated by ANME-1a and its partner *Ca.* Desulfofervidus auxilii (Krukenberg et al., 2018) Propane- and butane-oxidizing cultures produced from the same site were dominated by *Ca.* Syntropharchaeum butanivorans and *Ca.* Syntropharchaeum caldarium, respectively, both paired with *Ca.* D. auxilii (Laso-Pérez et al., 2016). In addition, we studied two ethane-oxidizing cultures. Both were derived from sediments collected during the AT37-06 expedition to the Guaymas Basin at a depth of 2001 m. E50 was maintained at 50 °C (Hahn et al., 2020), and E20 was maintained at 20 °C (Torset, 2025).

All cultures were produced and maintained as described by Laso-Pérez et al. (2018). Briefly, in an anaerobic chamber, the anoxic sediment was diluted with anoxic synthetic sulfate reducer medium (Widdel and Bak, 1992) at pH 7.2 (HR12, ISIS20) or at pH 6.5 for all other cultures. The resulting slurries were distributed into cultivation bottles and supplied with a gaseous alkane substrate (i.e., methane, ethane, propane, or butane). Cultures were incubated at the respective temperatures (Table 1). As a measure of substrate-dependent activity, sulfide concentrations were repeatedly measured in a photometric copper sulfate assay (Cord-Ruwisch, 1985). The medium was exchanged when sulfide values approached or exceeded 15 mM. Successive dilutions of the slurries (1:2 to 1:4) led to sediment-free enrichment cultures after 8 to 10 dilutions or 1–3 years. When alkane-dependent sulfide production rates exceeded approximately 250 μmol per liter per day, the sediment-free enrichments were diluted 1:2 to 1:4, depending on bottle size and activity. For this study, unless otherwise specified, sulfide was measured against a standard curve prepared from dilutions of a 25 mM standard, the concentration of which was routinely verified by iodometric titration (American Public Health Association, 1980).

### 2.2 Extraction of microbial metabolites from cultures

Sampling of polar metabolites was adapted from Sogin et al. (2019) with an added DCM lipid extraction simplified from Evans et al. (2022). Briefly, incubation bottles were inverted to allow the biomass to settle on or near the butyl rubber stopper. Then, 2 to 5 mL of the biomass was sampled using plastic syringes with large-gage (11G) hypodermic needles into 15 mL centrifuge vials. The samples were centrifuged, and the supernatant was transferred into another centrifuge tube. To extract polar metabolites from the biomass pellets, 2 mL of 1:1 water:MeOH was added. If samples were designated for quantification, the samples were spiked with ribitol (0.0714 mg mL^−1^) and cholestane (0.0286 mg mL^−1^). Samples were then sonicated using a Bandelin Sonoplus with an MS72 probe for 1 min at 97% power. The samples were then centrifuged at 3700 rpm, and the water:MeOH phase was transferred to 2 mL microcentrifuge tubes and stored at −20 °C until further treatment.

### 2.3 Derivatization of metabolites and gas chromatography–mass spectrometry analysis

Metabolites were analyzed using a modified version of the derivatization protocol described by Sogin et al. (2019). The water:MeOH sample extract was dried overnight using a rotary evaporator (Concentrator Plus, Eppendorf). After adding 250 μL of toluene and sonication, samples were dried again using a rotary evaporator (Concentrator Plus, Eppendorf). Toluene was evaporated at 37 °C at 250 rpm, and 80 μL of methoxyamine dissolved in dry pyridine (20 mg mL^−1^) was added to the dry sample, which was ultrasonicated for 10 min. Samples were then incubated at 37 °C for 90 min at 1350 rpm to evaporate the pyridine. Then, 100 μL N, O-bis(trimethylsilyl)trifluoroacetamide (BSTFA) was added. This mixture was sonicated and incubated at 37 °C for 30 min, and centrifuged at 13,000 rpm for 2 min.

The clear supernatant was analyzed using an Agilent 7890B GC coupled to an Agilent 5977A single-quadrupole mass-selective detector. 1 μL sample was injected via the GC inlet liner (ultra inert, splitless, single taper, glass wool; Agilent) onto a DB-5MS column (30 m by 0.25 mm, 0.25-μm film thickness, with a 10 m DuraGuard column; Agilent). The inlet liner was changed every 100 samples to prevent damage to the GC column and associated retention-time shifts. The injector temperature was set at 290 °C. Chromatography was performed with an initial column oven temperature set at 60 °C, followed by a ramp of 20 °C min^−1^ to 325 °C, then held for 2 min. Helium was used as the carrier gas at a constant flow rate of 1 mL min^−1^. Mass spectra were acquired in electron ionization mode at 70 eV across the mass range m/z 50–600 at a scan rate of 2 s^−1^. The retention time for the method was locked using a standard mixture of fatty acid methyl esters (Sigma-Aldrich). Peaks were annotated using Agilent Unknowns with peak filters of 28, 91, and 149, with area filters set to greater than or equal to absolute counts of 10. Deconvolution settings were an RT window size factor of 400, an extraction window of 0.3 m/z to the left and 0.7 m/z to the right, a sharpness threshold of 25%, a minimum number of ion peaks of 3, and a maximum number of peak shapes to store of 10.

### 2.4 Determination of metabolite-protein ratios

To quantify the total amount of soluble protein, extraction was performed as before, except using 5 mL of MeOH:water. Of these, 2 mL were used for metabolite extraction as before, and 2 mL were used for protein quantification with the Thermo Fisher MicroBCA kit according to the manufacturer’s instructions. The ratio R of metabolite *i* in sample *k* (μg metabolite per μg of protein) was then calculated as follows:


$$R_{k}^{i}=\frac{M_{k}^{i}(\mu g)}{P_{k}(\mu g)}=\frac{A_{k}^{i}\times m_{K}^{\text{IS}}(\mu g)}{A_{k}^{\text{IS}}\times WM_{k}(\mathrm{mg})\times [P_{k}](\mu g\;{\mathrm{mg}}_{\mathrm{WM}}^{-1})}$$

Where $M_{k}^{i}$ is the metabolite mass of metabolite *i* and $P_{k}$ is the soluble protein mass in sample *k,* respectively. $A_{k}^{i}$ and $A_{k}^{\text{IS}}$ are the peak areas of metabolite *i* and the ribitol internal standard in sample *k,* respectively. $WM_{k}(\mathrm{mg})$ is the total wet mass of sample *k* from which both the metabolite extraction and protein extractions were derived, and $\left[P_{k}\right]$ is the concentration of protein per mg of wet mass of the sample *k*. Each GC run was accompanied by a calibration mix composed of 2.5 μg of Lauric acid, 2-hydroxyhexanoic acid, Sucrose, Trehalose dihydrate, DL-Lysine, Leucine, Glycine, Di-sodium succinate hexahydrate, L-Ornithine monohydrochloride, DL-Serine, L-Asparagine, DL-Tryptophane, and Cholesterol.

### 2.5 Spectral matching of disaccharides

Spectral search for compound identification was performed against the NIST/EPA/NIH Mass Spectral Database (NIST 11; Stein et al., 1987), where match factors, scaled from 0 to 99, reflect the similarity between the observed spectrum and reference entries. Values above 90 are generally considered high-confidence matches. In addition, where available, metabolites were identified by comparison of retention times and fragmentation patterns with authentic standards. Trehalose was identified by matching both retention time and mass spectrum to an authentic trehalose standard and was correctly annotated by NIST 11. Diagnostic fragments included m/z 361, corresponding to a terminal silylated hexose unit [C_15_H_33_O_4_Si_3_]^+^, m/z 204 and 217, corresponding to a silylated pyranose ring, and m/z 191, corresponding to a fragment of silylated hexoses (Medeiros and Simoneit, 2007).

Tentative unknown disaccharides were evaluated by comparison with the authentic standards in the calibration mix above and a standard of 4-O-beta-galactopyranosyl-D-mannopyranose (GPMG). The unknown compounds discovered in this work produced fragments at m/z 361, 217, 204, 147, and 73, consistent with a glucose-containing silylated saccharide (Medeiros and Simoneit, 2007). In both unknowns discussed in this work, the m/z 361 glucose-associated fragment had a substantially lower intensity than the m/z 204 fragment compared to trehalose. The unknowns discussed in the text below were further characterized by the m/z 204 and 217 ratios, which were nearly identical between the two compounds, indicating similar fragmentation patterns despite their different elution times. The additional m/z 351 fragment was observed in the unknown compounds (red arrow) but not in authentic GPMG or any other disaccharide standard. Due to the presence of other fragments in addition to the diagnostic peaks of saccharides, different fragment ratios, and different elution times, the NIST-recommended annotations of the unknowns discussed in this work were rejected.

### 2.6 Curation and annotation *Halobacteriota* and *Desulfobacterota* genomes from the GTDB

The GTDB (R220; Parks et al., 2022) accessions of the phyla *Halobacteriota* and *Desulfobacteriota* were fetched with the gtt-get-accessions-from-GTDB command from the GTotree (v1.8.3) pipeline (Lee, 2019). This dataset was further curated using a custom Python script with ANME-1 and ANME-2 defined as in Chadwick et al. (2022) and Laso-Pérez et al. (2023), *Ca.* Ethanoperedens as defined by Chen et al., 2019 and Hahn et al. (2020), *Ca.* Syntropharchaeum, as defined by Laso-Pérez et al. (2016), *Ca.* Alkanophaga, as defined by Zehnle et al. (2023), and *Ca.* Methanoliparia is defined by Laso-Pérez et al. (2019). ANME-3 were not included. In addition, a custom subset of non-ANKA *Halobacteriota* was randomly selected, with the criterion that no individual genus could be represented by more than five entries to prevent overrepresentation of lab strains of the *Halobacteriales*. The accessions for these genomes (Supplementary Table S5) were downloaded from NCBI using *datasets* (v.16.43.0) (O’Leary et al., 2024) with the flags *--download genome accession “${sample}” --include gff3,rna,cds, protein, genome,seq-report,* where ${sample} refers to the specific accession number. The downloaded MAGs were then annotated with Prokka (v. 1.14.6) (Seemann, 2014) with the *flags --kingdom K --force --centre X –compliant* where K is Archaea or Bacteria depending on the run. The completeness and contamination of the genomes was assessed with CheckM2 *predict* (v1.0.2) (Chklovski et al., 2023) with the flags *--threads 2 --genes --input $protein –force* where $protein is the amino acid file of the corresponding MAG generated by Prokka in the previous step.

For *Desulfobacteria*, all GTDB entries assigned to the Seep-SRB-1a, −1d, and -1 g clades, the Seep-SRB2 clade, and the class *Desulfofervidia* were selected, according to Schreiber et al. (2010) and Murali et al. (2023). In addition, a curated subset of *Desulfovibrionia*, *Desulfuromonadia*, and *Desulfobacterales*, excluding the known ANKA partner bacteria, was randomly selected using the same genus criteria as above, along with the additional criterion that no single order accounts for more than 20% of the total dataset. This prevented the overrepresentation of the very abundant *Desulfovibrionales* genomes. Accessions for all genomes analyzed in this study, along with their GTDB metadata, are provided in Supplementary Table S5.

### 2.7 Annotation of genes for disaccharide metabolism and protein phylogenies

A set of curated HMMs of various protein families associated with trehalose, sucrose, and general disaccharide metabolism was downloaded from InterPro (v.104.0) (Blum et al., 2025). The profiles used, their parent families, and associated functions are listed in Table 2. These were searched against the curated sets of GTDB genomes using *hmmsearch* (v.3.4) (Eddy, 2011). The *hmmsearch* outputs were parsed using a custom Python script, and hits were filtered for E-values ≤1e-10 and hmm coverage ≥40%. To prevent cross-annotation, each locus tag was only considered for its best hit. The average HMM hits per genome (*λ*) for each taxonomic group was calculated as:


$$\lambda_{G}=\frac{1}{N_{G}}\;\sum_{i=1}^{N_{G}}H_{i}$$

**Table 2:** Protein families of HMM profiles used in this study.

Name	Family	KEGG orthology	Summary of Interpro entry
TreA (TreF)	PF01204	K01194/K22934	Reversible hydrolysis of alpha-alpha trehalose into beta and alpha glucose (Mori et al., 2009).
TreH (N-Terminal)	PF19291	K01194/K22934	Trehalase-like domain characterized in Sulfolobus (Moon et al., 2016)
TreP/MalP (GH65)	PF03632	K22248	Subset of Cazy family GT4—reaction is reversible and, unlike the hydrolases, maintains the alpha structure of both glucose monomers.
TreZ	TIGR02402	K01236	Free trehalose-forming/consuming component of the reversible tre(X)YZ machinery, which ties trehalose to polysaccharides (Moon et al., 2016).
TreT (N-Terminal)	PF21269	K13057	Catalyzes the formation of α-trehalose from Nucleoside Diphosphate glucose (i.e, ADP or UDP glucose) and glucose. It is reversible in *P. hoirikoshi* but not in *T. tenax* (Kouril et al., 2008; Woo et al., 2010).
TreS (Alpha Amylase Domain)	PF00128	K05343	The polysaccharide to maltose component of the TreS.
TreS (Maltogenic domain)	PF16657	K05343	Maltogenic domain of the TreS.
OtsA (GT20)	PF00982	K00697	Irreversible synthesis of Trehalose from UDP glucose and Glucose-6-P.
Sus	TIGR02480	K00695	Reversible synthesis of sucrose from U(A)DP-glucose and D-fructose.
Sps	PTHR46039	K00696	While, in principle, the reversible formation of Sucrose-6-Phosphate from fructose-6-phosphate and UDP-glucose is possible, it is usually coupled to SPP for unidirectional synthesis.
Spp	TIGR01485	K07024	Irreversible hydrolysis of sucrose-6-phosphate to sucrose drives the reaction toward sucrose synthesis.
sacB	PF02345	K00692	Reversible synthesis of the fructose polysaccharide levan from sucrose, yielding one free D-glucose.
sucP	PIRSF003059	K00690	Contains both reversible sucrose phosphorolysis to fructose and alpha-Glucose; it also contains Glucosyl glycerate phosphorylases, which are not specific to sucrose.
GT1	PF00534	NA	Diverse carbohydrate linkers make EPS; lipopolysaccharides, among other targets.
GT4	PF00953	NA	Diverse family involved in glucose transfer, but also includes some sucrose synthases; the only characterized function in archaea is trehalose synthases.
GT2	PF00535	NA	Diverse family, but few are characterized in archaea; known to be involved in cell-wall biosynthesis; various glycosylation reactions, but also chitin, cellulose, and sucrose biosynthesis.
GH13	PF00128	NA	Diverse family of glycosyl hydrolases; most characterized functions are either debranching, synthesizing, breaking down, or otherwise remodeling glucose-based polymers.
GH57	PTHR3606	NA	Diverse family—is well described in hyperthermophilic archaea and acts as a glucose polymer degrader.

Where $N_{G}$ is the number of genomes in the taxonomic group G, and $H_{i}$ is the number of HMM hits for protein family *i* in the group G.

Because TreT, TreP/MalP, and OtsA coding genes are widespread in ANKA and partner bacteria, their amino acid sequences were phylogenetically analyzed. We extracted the sequences of locus tags with valid HMM hits from the Prokka annotations of the genomes. For each HMM profile, only the locus tag associated with the best hit was considered. These were then concatenated and aligned using MAFFT-Linsi (v7.520) (Katoh et al., 2005; Katoh and Standley, 2013) and trimmed using trimAL (v1.4) (Capella-Gutiérrez et al., 2009) with the *-gappyout* option. The trimmed alignments were passed to IQtree2 with the automatic model finder and 1,000 ultrafast bootstraps (Kalyaanamoorthy et al., 2017; Minh et al., 2020). All trees were visualized and edited with iTOL (v7) (Letunic and Bork, 2024). We defined an “instance” of horizontal gene transfer (HGT) as a case in which the closest homolog, or closest group of homologs, to a protein or protein clade conflicted with the phylogenetic genome taxonomy for ANKA and SRBs (Knittel et al., 2026). Such conflicts are most apparent for interdomain gene transfer (Archaea vs. Bacteria). For example, if the protein sequence of a single ANKA group clusters with sequences from bacteria, a HGT event from bacteria to archaea may have occurred.

### 2.8 Assimilation of ^13^C from ^13^C-DIC and ^13^C-trehalose into bacterial and archaeal lipids

We assessed the dependence of CO_2_ assimilation and trehalose assimilation on alkane availability using the available E50 culture, which is an ethane-oxidizing enrichment dominated by *Ca.* Ethanoperedens thermophilum (Hahn et al., 2020). We washed the E50 biomass from two 700-mL bottles with clean, non-labeled medium. We transferred 25 mL of dense E50 biomass into replicate 150-mL culture bottles, which we filled to 100 mL with 10 mM bicarbonate dissolved in inorganic carbon (DIC)-containing culture medium.

For the ^13^C-DIC labeling experiment, six subcultures of E50 received 100 μL of a ^13^C-DIC standard solution (1 M NaH13CO3; Sigma, in MilliQ water), yielding δ^13^C-DIC values of +550‰. These subcultures were split into two sets of three biological replicates; half of the replicates were incubated with ethane headspace, and the other half did not receive a gaseous energy source. Sulfide was sampled after 0,3,6,9, and 24 days of incubation (Supplementary Table S7), and the first and last time points were sampled for lipids and DIC analyses.

For the ^13^C-trehalose experiment, an anoxic stock of 20 mg mL^−1^ 5% ^13^C-labeled trehalose was prepared by adding 10 mg of ^13^C-labeled trehalose (Sigma-Aldrich) and 190 mg of non-labeled trehalose (Merck) to 10 mL of anoxic water (δ^13^C_trehalose_ ≈ 9,800‰). The stock was sterile-filtered (0.2 μm, Minisart, Sartorius) and stored under oxygen-free conditions. Due to the limited availability of E50 culture biomass, we prioritized time-series resolution over the number of replicates. As described above for the DIC incubation, 1 L subcultures of E50 were prepared; 10 subcultures received 100 μL ^13^C-trehalose stock solution, and 1 control was kept trehalose-free. The 10 trehalose-containing subcultures were then split into two experimental sets of five. One set received ethane as an energy source; the other half received an N_2_: CO_2_ gas mixture. The cultures were incubated at 50 °C for 15 days; at 0, 3, 6, 9, and 15 days, sulfide was measured from all remaining bottles, and one culture bottle was sacrificed for lipid extraction in each experimental condition (Supplementary Table S8).

For the extraction of lipids, the procedure for metabolite extraction was modified to use a water:methanol:dichloromethane (DCM) (1:1:2) mixture as the solvent. The samples were sonicated in a Bandelin Sonorex water bath and rested on ice to allow phase separation. The resulting DCM phase was transferred to microcentrifuge tubes, evaporated to dryness and stored at −20 °C until further treatment.

One-third of the TLE was saponified with 6% potassium hydroxide (Sigma-Aldrich) in methanol (MeOH, Sigma-Aldrich) for 3 h at 80 °C. The product was extracted three times with n-hexane to remove neutral lipids (NL). After that, the sample was acidified with HCl, and the fatty acids (FA) were extracted with n-hexane. The FA fraction was dried and derivatized with 10% BF_3_ in MeOH (VWR) at 70 °C for 1 h to form fatty acid methyl esters (FAMEs), which were then extracted with hexane. To yield the isoprenoid hydrocarbons from archaeal ether lipids, one-third of the TLE was subjected to ether cleavage using the boron tribromide treatment, followed by reduction with lithium triethylborohydride (Summons et al., 1998).

Analyses of FAMEs and ether-cleaved isoprenoids were carried out using gas chromatography (GC) coupled to flame ionization detection (Trace GC Ultra, Thermo Scientific). GC cross-checked compound identities using mass spectrometry (Trace 1,310 + ISQ 700, Thermo Scientific) operated in EI mode at 70 eV and collecting full-scan spectra from m/z 50 to 850. Stable carbon isotope compositions (expressed as δ^13^C values relative to Vienna PeeDee Belemnite standard, VPDB; Coplen, 1994) were determined using GC–isotope ratio mass spectrometry (Trace GC Ultra connected with a GC-IsoLink and interfaced through a ConFlo IV to a Delta V Plus IRMS, Thermo Scientific). All GC analyses were performed in splitless mode (1 min at 310 °C) and used an identical Restek Rxi-5 ms column (30 m × 0.25 mm ID × 0.25 μm, Restek). The temperature program for all instruments was: 60 °C for 1 min, followed by heating to 150 °C at 10 °C min^−1^, then to 310 °C at 4 °C min^−1^, with a final hold at 310 °C for 30 min for the previously cleaved isoprenoids from the archaeal lipids, and 20 min for FAMEs. Helium served as the carrier gas at 1.0 mL min^−1^, except during GC–IRMS runs where 1.2 mL min^−1^ was used. For the latter analyses, compounds eluting from the column were oxidized to CO_2_ in a combustion reactor maintained at 940 °C. The instrument was calibrated against a CO_2_ reference gas with a known isotopic composition, and precision was monitored by repeated analysis of a C20–C40 n-alkane mixture, which exhibits long-term variability of ±0.5‰. δ^13^C values of FAMEs were corrected for the added carbon introduced during derivatization. The isoprenoids phytane and biphytane were considered diagnostic of *Ca.* Ethanoperedens and the straight-chain fatty acids C16:0 and C18:0 were considered diagnostic for its partner SRB, *Ca.* D. auxilii, while iso-branched fatty acids *i*15:0, *i*16:0, and *i*17:0 were considered diagnostic for ancillary community heterotrophs (Stock et al., 2025; Zhu et al., 2022).

### 2.9 Use of artificial intelligence

The authors declare that they used AI-empowered tools like Grammarly and ChatGPT to support the writing of this manuscript. The authors also used AI services, primarily ChatGPT, to debug code and assist in generating code for this project. The validity of code and text was verified by the responsible author.

## 3 Results

### 3.1 Disaccharides are abundant in alkane-oxidizing enrichment cultures

**Figure 1:**
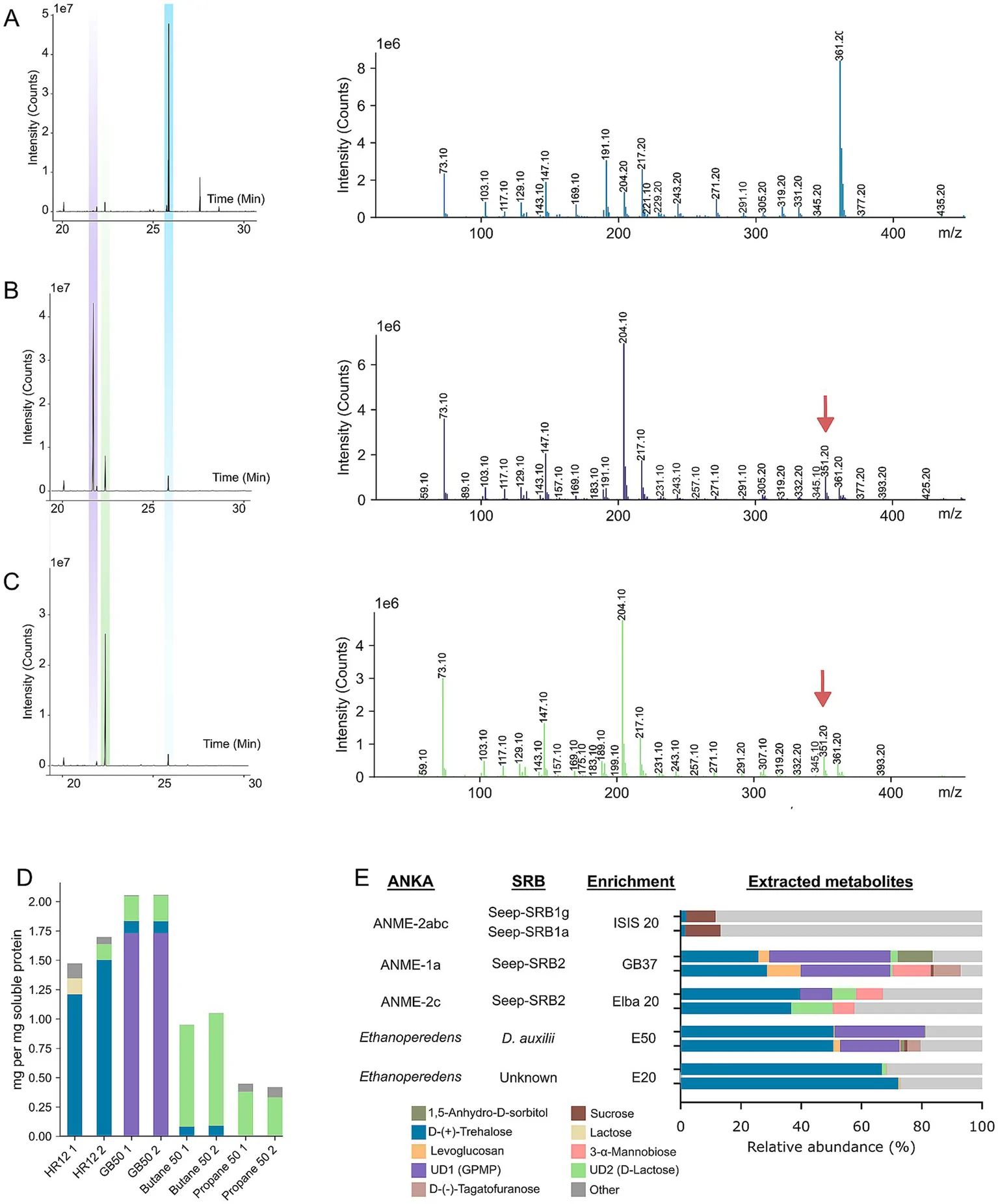
Extracted disaccharides and their mass spectra in alkane-oxidizing enrichments. **(A–C)** 20–30 min section of the chromatogram of the HR12, GB50, and Butane50 cultures and mass spectra of their most abundant disaccharide. **(A)** Blue: Trehalose at RT 25.86 min, **(B)** Purple: Unidentified disaccharide isomer 1 at RT 21.70 min; **(C)** Green: Unidentified disaccharide isomer 2 (RT 22.36). Compound identification was performed using NIST Mass Finder 1.1, with match factors of 97 and 87 for trehalose and lactose, respectively. Each sugar is annotated with an octakis(trimethylsilyl), which has been removed for clarity. The *m/z* 351 discussed in the text is marked with a red arrow. **(D)** Stacked bar-chart of disaccharide concentration in mg disaccharide per mg soluble protein in select alkanotrophic enrichment cultures. The bars are shaded according the legend in **(E)**. **(E)** Relative concentrations of extracted polar metabolites. The bar chart is labeled with the host enrichments and their constituent ANME/SRB consortia members. Highlighted in colors are sugars of particular interest as annotated by NIST, with the -silyl prefixes removed for clarity.

Here, we analyzed metabolites from sediment-free methane-, ethane-, propane-, and butane-oxidizing enrichment cultures derived from multiple marine gas-rich sediments enriched in different ANKA-SRB consortia spanning methane to butane oxidation and a temperature range of 12 to 50 °C (Table 1). GC–MS analysis of small polar metabolites showed that BSTFA-derivatized disaccharides represented more than 50% of the annotated metabolite profiles of all enrichments cultures except the ANME-2a/2b/2c-dominated ISIS20 (Figures 1A,E) alongside lower contributions from amino acids such as glutamate and aspartate (Supplementary Tables S1, S2).

In the HR12 culture, the dominant disaccharide was identified as trehalose, based on retention time and spectral matching (see Methods). Trehalose reached 1.5–2 mg per mg of soluble protein as assessed using the microBCA assay (Supplementary Tables S2–S4). In contrast, the ANME-1-dominated GB50 culture contained only 0.2 mg trehalose per mg of soluble protein (Figure 1D) and the metabolite extract was dominated by an unidentified compound that elutes at 21.7 min and demonstrated a spectral match to a hexose containing disaccharide (Figures 1B,D, see Methods). In the propane- and butane oxidizing cultures Propane50 and Butane50, similar compounds eluted at 22.4 min with an almost identical spectra (Figures 1B,C). Although this peak was annotated as 4-O-beta-galactopyranosyl-D-mannopyranose (GPMG) with a match factor of 80 (Supplementary Table S3), authentic GPMG standards eluted later and lacked the 351 m/z fragment detected in the GB50, Butane50, and Propane50 compounds (Figures 1B,C; Supplementary Figure S1), suggesting that they produce unknown compounds.

We screened other AOM cultures for the presence of disaccharides. All low-temperature AOM cultures (Elba 20, HR12, GB37) contained trehalose (Figure 1E; Supplementary Table S1). The ANME-1a-dominated GB37 enrichment contained substantial amounts of trehalose and the unknown disaccharide initially annotated as GPMG described above. The unknown disaccharide was also present in the 50 °C ethane-oxidizing enrichment culture E50 dominated by *Ca.* Ethanoperedens and *Ca.* D. auxilii, whereas the room-temperature ethane-oxidizing E20 enrichment culture, contained almost exclusively trehalose. The ANME-2a/2b-dominated ISIS20 culture was unique because sucrose was the dominant disaccharide (Figure 1E).

### 3.2 Some ANKA and associated SRB’S encode complementary disaccharide metabolism

**Figure 2:**
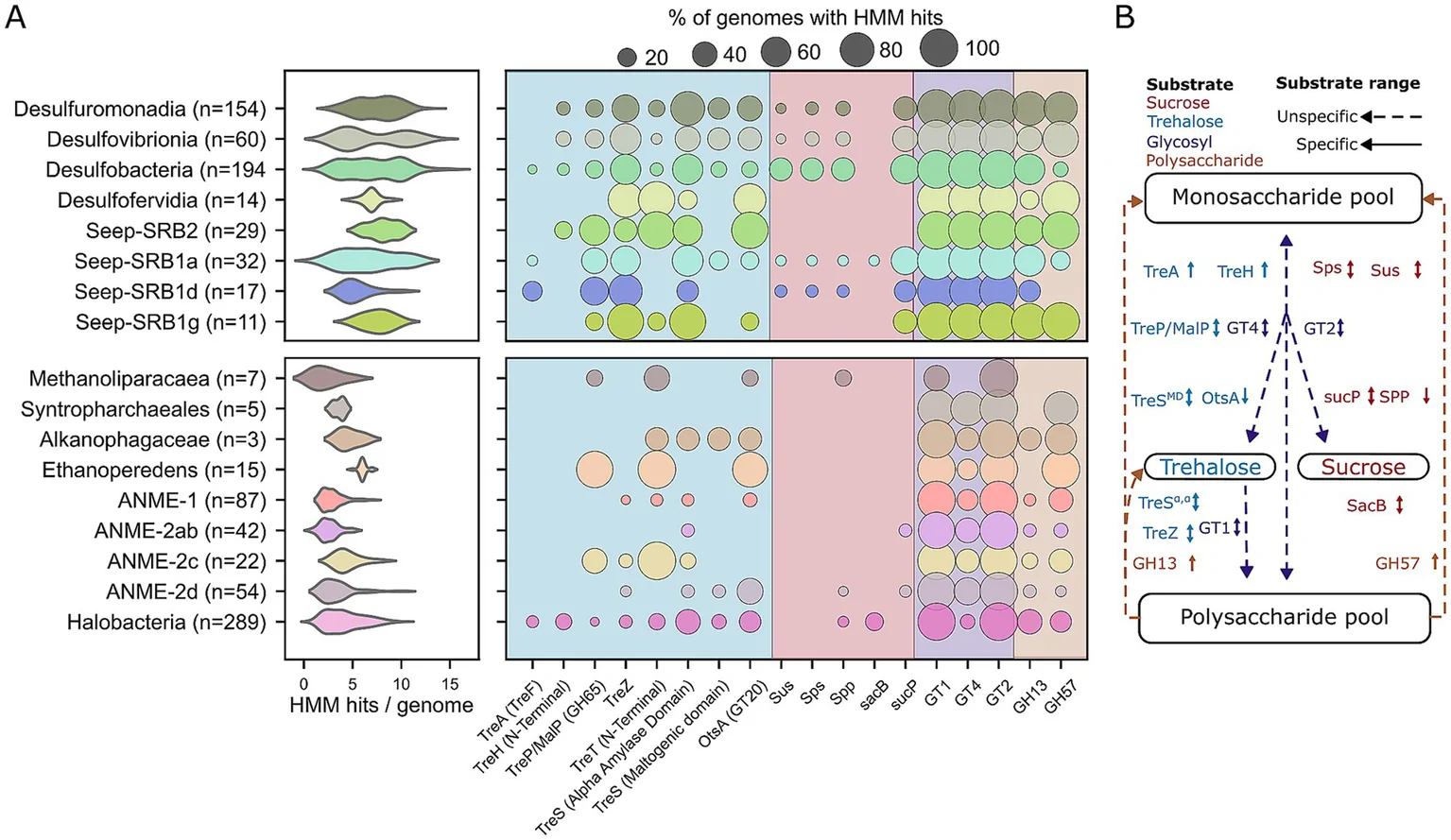
Distribution of select disaccharide proteins in the genomes of ANKA, partner SRBs, and reference organisms. **(A)** HMM hits of proteins in trehalose-, sucrose-, and related carbohydrate metabolism across GTDB (R220)–deposited genomes of ANKA and known SRB partners, as well as selected *Desulfobacterota* and non-ANKA *Halobacteriales* genomes according to HMM profiles. Hits were retained for e-values ≤1e-10 and HMM profile coverage ≥40%. Each gene was assigned to only one HMM profile. The left panel shows the number of hits per genome for each taxonomic group with *n* number of MAGs displayed with the name of the corresponding group; the central panel shows the percentage of genomes in each group that contain at least one hit associated with the queried HMM profiles. Blue indicates reactions directly operating on trehalose, red indicates sucrose, and purple indicates glycosyl transferase families that may operate on trehalose or sucrose. In contrast, gold indicates select glycosyl hydrolases that degrade polysaccharides. **(B)** Schematic overview of known enzymes for disaccharides trehalose (blue) and sucrose (red) metabolisms, as well as select polysaccharide degradation (gold) and glycosylation metabolisms (purple) that intersect with the carbohydrate pools investigated in this study. Enzymes with specific substrates are shown with solid lines, while enzymes with broad substrate ranges are shown with dashed lines. Above each enzyme name is an arrow that indicates its known directionality. The enzymes used for HMM profiles are listed in Table 2. TreA (TreF), trehalase; TreH, reversible *α*,α-trehalose hydrolase; TreP/MalP, trehalose/maltose phosphorylase; TreZ, trehalose-6-phosphate hydrolase; TreT, trehalose glycosyltransferase; TreS, maltose-forming trehalose isomerase; OtsA, trehalose-6-phosphate synthase; Sus, sucrose synthase; Sps, sucrose-phosphate synthase; Spp, sucrose-phosphate phosphatase; SacB, levansucrase; SucP, sucrose/glucosylglycerate phosphorylase; and broad glycosyltransferase (GT1, GT2, GT4) and glycoside hydrolase (GH13, GH57) families.

We searched ANKA, partner SRB, and reference *Halobacteriota*/*Desulfobacterota* genomes in a genomic database constructed from MAGs deposited in the GTDB (R220) for HMM profiles associated with trehalose, sucrose, and polysaccharide metabolism (Methods, Table 2; Supplementary Table S5). Among the ANKA lineages, *Ca.* Ethanoperedens showed the highest potential for disaccharide metabolism with an average of 6.1 HMM hits per genome (*λ* = 6.1, Figure 2). *Ca.* Alkanophagaceae also had a substantial average number of hits for disaccharide metabolism, but this average is based only on three deposited genomes (λ = 4.7, *n* = 3). ANME-2c (λ = 4.5, *n* = 22) also generally encoded multiple key enzymes, including trehalose synthase (TreT), trehalose-maltose phosphorylase (TreP/MalP), and trehalose-6-phosphate synthase (OtsA). These values are typical for *Halobacteriota* (λ = 4.1, *n* = 289). Other ANME groups, including ANME-2a/2b (λ = 2.6, *n* = 42), ANME-2d (λ = 3.3, *n* = 54), and ANME-1 (λ = 2.9, *n* = 87), have, on average, far fewer HMM hits. Lower λ values were also observed in the butane and propane oxidizers of the *Ca.* Syntropharchaeales (λ = 3.6) and the long-chain alkane oxidizers of the *Ca.* Methanoliparaceae (λ = 2.1), although this average is based on few deposited MAGs (*n* = 5 and 3, respectively; Figure 2). Variants that catalyze the irreversible degradation of trehalose are absent in all ANKA.

In comparison, SRB partner clades showed broader and more variable disaccharide-metabolism profiles. In SRB partners, λ ranged from 5.5 in Seep-SRB1d to 8.1 in Seep-SRB2. Several SRB clades known to engage in syntrophic partnerships with ANKA, such as Seep-SRB1a and Seep-SRB2, contained MAGs with HMM hits to the irreversible trehalases TreH and TreA (Figures 2A,B). However, in both cases fewer than half of all the queried MAGs had trehalase hits. Seep-SRB1a resembled the broader distribution of *Desulfobacteria*, while other classes of the *Desulfobacterota*, like *Desulfovibrionia* and *Desulfuromonadia*, displayed bimodal distributions of HMM hits per genome, indicating that there is likely sub-class level differentiation in disaccharide metabolism (Figure 2).

### 3.3 ANKA and their partner bacteria share trehalose metabolism via horizontal gene transfer

To investigate if the trehalose metabolism in ANKA and SRB were vertically inherited or if the widespread presence in the genomes is the result of repeated horizontal gene transfer, we constructed amino-acid sequence-based phylogenic trees for three key genes involved in trehalose biosynthesis and/or degradation: trehalose-6-phosphate synthase (OtsA), trehalose/maltose phosphorylase (TreP/MalP), and trehalose glycosyl-transferring synthase (TreT).

**Figure 3:**
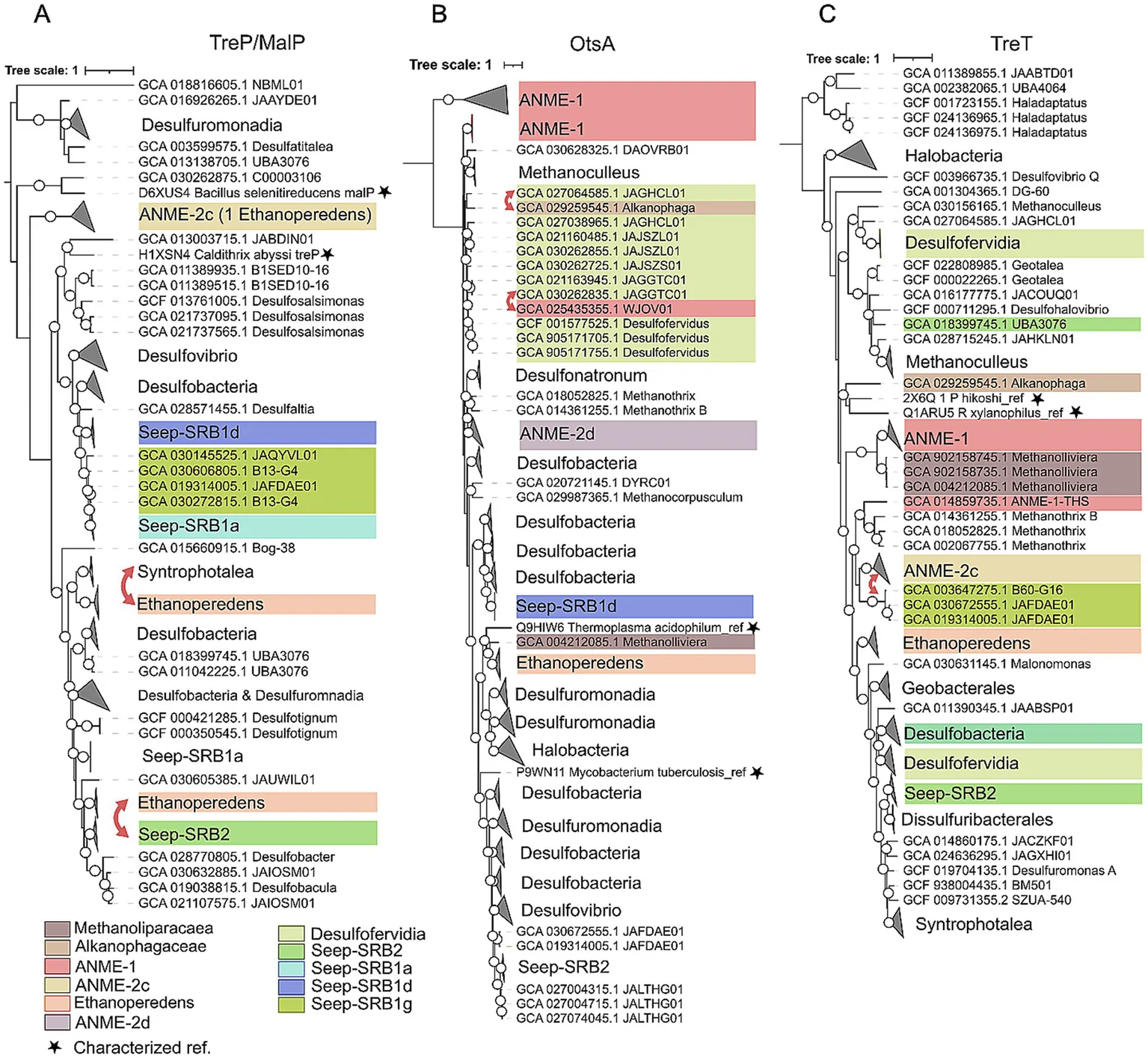
Protein phylogenetic trees of trehalose-metabolizing enzymes OtsA, TreP/MalP, and TreT in ANKA and SRB partners. Protein trees of best-hit locus tags to the HMMs corresponding to the protein families of **(A)** TreP/MalP (GH65), **(B)** OtsA (GT20), and **(C)** TreT. The branches are shaded according to their membership in an ANKA or SRB partner functional group, as in Figure 2, and proteins with characterized references are shown in purple. Red arrows mark potential ANKA/SRB HGT events. In all trees, bootstraps greater than 90% are shown as white circles. Grey stars show characterized references, and functional and taxonomic groupings of interest are highlighted by boxes following the color scheme of Figure 2.

TreP/MalP is common in *Ca.* Ethanoperedens, ANME-2c, and most SRB partner clades, but is rare in other ANKA. The majority of the hits retrieved were more similar to a characterized trehalose phosphorylase than to a characterized maltose phosphorylase (Figure 3A). The TreP/MalP coding sequences of ANME-2c form a separate phylogenetic clade with a single member of *Ca.* Ethanoperedens, indicating HGT between these two ANKA lineages (Figure 3A). The TreP/MalP sequences of the remaining *Ca.* Ethanoperedens genomes form two distinct phylogenetic clusters. One cluster, including *Ca.* Ethanoperedens thermophilum, shares ancestry with the TreP/MalP proteins of Syntrophotalea, while *Ca.* Ethanoperedens ethanivorans TreP/MalP proteins share an ancestor with those of Seep-SRB2. Similarly, the TreP/MalP-encoding sequences of Seep-SRB1a are separated into two phylogenetic clades: one is phylogenetically distinct, and the other is shared with Seep-SRB1g and SRB1d and appears to be rooted in a shared protein ancestor with *Desulfovibrio*.

OtsA, a trehalose-6-phosphate synthase, catalyzes the irreversible conversion of UDP-glucose to trehalose and is found in representatives of all three domains of life (Avonce et al., 2006). In our metagenomic dataset, OtsA sequences separated into two distinct clades, one of which is composed exclusively of ANME-1 (Figure 3B). One of these clades is a cluster of four proteins, all annotated as D-inositol-3-phosphate glycosyltransferases, that fell at the lower end of our similarity selection criteria (Supplementary Table S6). Beyond these, only six of the 95 *Ca.* Syntropharchaeales genomes contain an OtsA homolog. Two of these represent clear cases of HGT involving ANME-1 and *Ca.* Alkanophaga genomes (Figure 3B), as these archaeal proteins are nested in an otherwise bacterial clade. In both cases, the closest OtsA homologs were from their SRB partners, the *Ca.* Desulfofervidia. On the other hand, ~45% of ANME-2d genomes and all but two *Ca.* Ethanoperedens genomes encoded OtsA homologs (Figure 2B; Supplementary Table S5). However, phylogenetic reconstruction of OtsA revealed that these closely related ANKA groups acquired *otsA* independently (Figure 3B). The ANME-2d sequences form a clade with *Methanothrix*, consistent with vertical inheritance from a shared ancestor. *Ca.* Ethanoperedens and one MAG of *Ca.* Methanolliviera seemed to have acquired *otsA* from a common ancestor shared with the *Desulfuromonadia*. *otsA* appears to have been acquired twice independently within the *Desulfuromonadales*, with one clade of OtsA sharing an ancestor with *Ca.* Ethanoperedens and the other clade appear to preserve bacterial phylogeny because they share a root with the characterized *Mycobacterium* reference. A second *Desulfuromonadales* group is nested within the *Desulfobacterales* in the OtsA tree, suggesting HGT from within that order.

TreT catalyzes the reversible formation of trehalose from glucose (Table 2) and also showed signs of interdomain HGT. TreT sequences encoded by *Desulfofervidia* formed two distinct phylogenetic clades (Figure 3C). One of these clades appears to root with the hydrogenotrophic genus *Methanoculleus*, suggesting HGT between SRB and non-ANKA archaea. The second TreT clade of the *Desulfofervidia* is nested within a clade with other SRB partners and non-partner SRB, and this clade shares an ancestor with *Ca.* Ethanoperedens.

### 3.4 Stable isotope probing reveals activity of trehalose-consuming side communities

**Figure 4:**
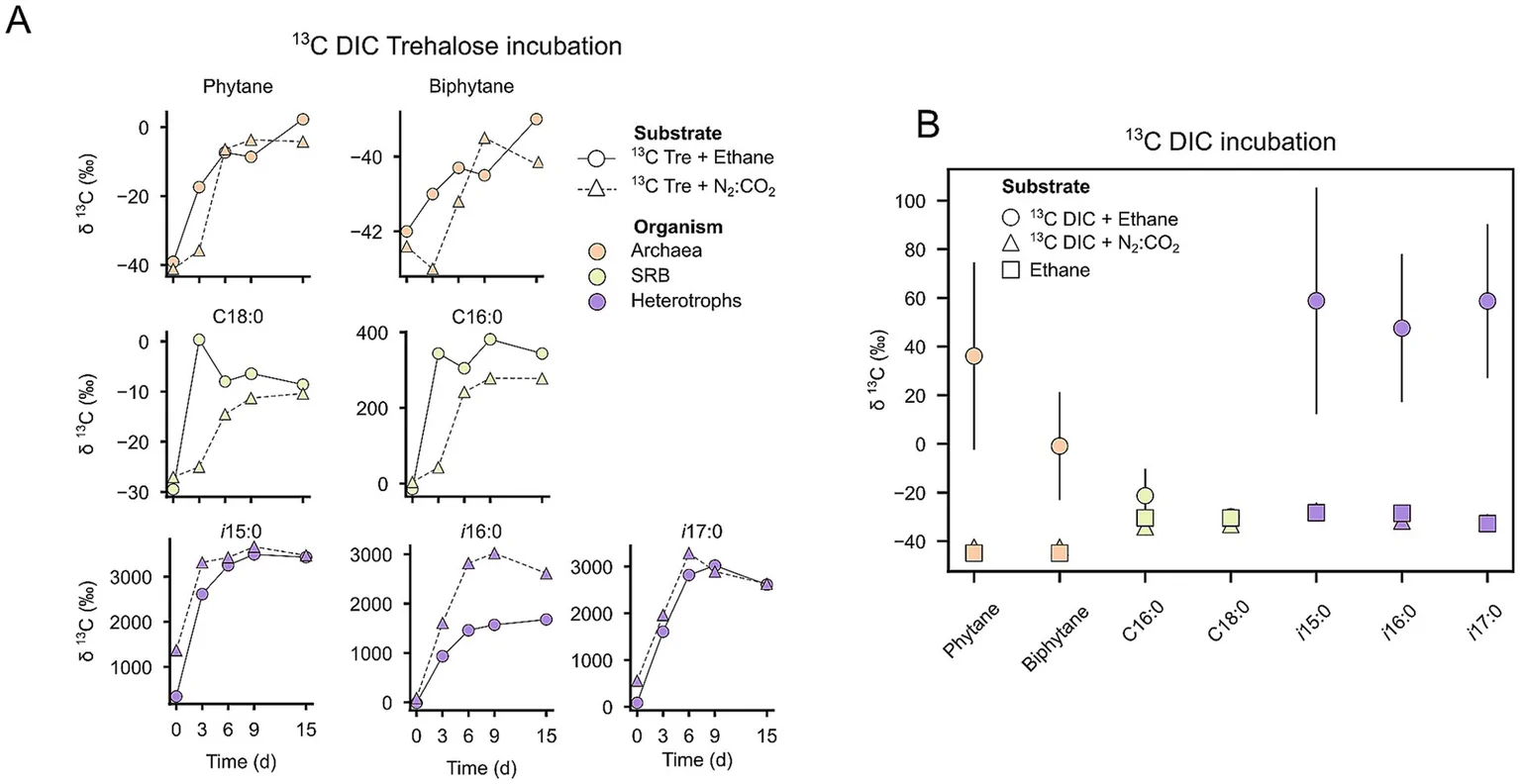
Incorporation of ^13^C-trehalose and ^13^C-DIC into bacterial and archaeal lipid moieties, in the E50 culture with and without ethane. **(A)** Shift in ^13^C values of marker lipids signaling heterotrophs (purple), SRB (light-green), and ANKA (light-orange) during trehalose incorporation over 15 days. **(B)** Change of δ^13^C values of the same lipids during DIC incorporation after 24 days. Data points represent the mean of triplicate incubations, with standard deviations visualized as error bars. For both experiments, activity was tracked by sulfide formation (Supplementary Tables S7, S8).

We investigated the role of trehalose in the syntrophic interactions within the ethane-oxidizing E50 enrichment by tracking ^13^C-trehalose incorporation into diagnostic lipids of its constituent microbes: isoprenoid hydrocarbons phytane and biphytane for the ANKA *Ca.* Ethanoperedens thermophilum, straight-chain fatty acids C16:0 and C18:0 for its SRB partner *Ca.* D. auxilii, and iso-branched fatty acids *i*C15:0, *i*C16:0, *i*C17:0 for ancillary community heterotrophs. Trehalose was selected due to its high concentration in the E50 enrichment culture (Figure 1) and the abundant enzymatic machinery of both the constituent ANKA (*Ca. E. thermophilum*) and SRB partner (*Ca.* D. auxilii) for trehalose metabolism (Figure 2). E50 culture aliquots were incubated with approximately 10% ^13^C-labeled trehalose, either with or without unlabeled ethane, and sampled over 15 days (Figure 4A). Incubations of E50 with trehalose showed similar sulfide formation and δ^13^C-DIC values = 283‰ without ethane and 289‰ with ethane, indicating substantial organoheterotrophic trehalose consumption (Supplementary Table S9; Supplementary Figures S2–S4). The ^13^C-incorporation into lipids was tracked to identify the organisms assimilating trehalose-derived carbon. The strongest assimilation of ^13^C-trehalose was measured in the heterotroph-associated iso-branched fatty acids *i*C15:0, *i*C16:0, and *i*C17:0, which reached δ^13^C-values >1,000‰ within hours and >3,000‰ within 7 days. Fatty acids primarily associated with the SRB partner showed lower incorporation of trehalose-derived carbon (δ^13^C-C16:0 < 400‰, δ^13^C-C18:0 < 30‰; Figure 4A; Supplementary Table S10). The C16:0 fatty acid is also potentially produced in part by heterotrophs; hence, the higher δ^13^C values could be attributed to heterotrophic production (Heinzelmann et al., 2015). The small change in δ^13^C for C18:0 may also be a result of secondary DIC uptake, as ^13^C-labeled carbon is readily transferred into the DIC pool. The archaeal markers of *Ca.* Ethanoperedens phytane (δ^13^C < 10‰) and biphytane (δ^13^C < −40‰) showed minor, ethane-independent, ^13^C incorporation, although it was an order of magnitude below the transfer to the DIC pool.

To assess the ethane dependency of the heterotrophs in the E50 enrichment on the activity of *Ca. E. thermophilum* consortia, we incubated aliquots of the E50 culture with ^13^C-DIC, either with or without ethane as the only supplied energy source (Figure 4B; Supplementary Figures S5–S7; Supplementary Table S11). The incorporation of DIC into the isoprenoids produced by *Ca.* Ethanoperedens depended on ethane (δ^13^C-phytane = 36 ± 39‰, δ^13^C-biphytane = −1 ± 22‰; Figure 4B). Likewise, ^13^C-DIC assimilation into the fatty acids predominantly produced by the partner SRB depended on the presence of ethane, although incorporation into these lipids remained low (δ^13^C-C16:0 = 11 ± 11‰, δ^13^C-C18:0 = 18 ± 22‰). On the other hand, heterotroph-associated branched fatty acids showed stronger DIC-derived ^13^C incorporation (δ^13^C-*i*C15:0 = 59 ± 47‰, δ^13^C-*i*C:16:0 = 48 ± 30‰, δ^13^C-*i*C17:0 = 59 ± 30‰).

## 4 Discussion

This study demonstrates the accumulation of substantial amounts of trehalose and other disaccharides in sediment-free, alkane-oxidizing enrichments. Together with the distribution and evolutionary history of disaccharide metabolism in ANKA and their SRB partners, this soluble disaccharide pool suggests that carbohydrate metabolism plays a hitherto unexplored role in anaerobic alkane oxidation and thus in sedimentary carbon turnover. However, which organism in enrichments composed of ANKA, SRBs and other community members is the source of the disaccharides, as well as the physiological functions of these compounds, remain unresolved.

### 4.1 Disaccharides are abundant in ANKA enrichments, but their source remains unknown

In cold-adapted methane-oxidizing and cold- and hot-adapted ethane-oxidizing cultures, trehalose is abundant, reaching up to 1.5–2 mg trehalose per mg soluble protein (Figure 1). This is almost twice the highest levels reported so far for the archaeon *Haladaptatus paucihalophilus* when cultured under extreme osmotic stress (Youssef et al., 2013), but within the highest ranges reported for halophilic SRB responding to salt stress (Welsh et al., 1996). In other cultures, such as GB50 or Butane50 from the Guaymas Basin, other, yet-unidentified disaccharides are more abundant. The shared m/z 351 signal, similar but distinct elution times, and glucose-containing fingerprints of these compounds indicate that they are likely isomers. The unknown metabolites were dominant only in the thermophilic cultures, all of which contain members of the *Syntropharchaeia* (i.e., ANME-1, *Ca.* Syntropharchaeum). Future studies may include other alkane-degrading cultures (hexane- to dodecane-oxidizing cultures containing *Candidatus* Alkanophaga) (Zehnle et al., 2023) or other thermophilic ANME-1-containing AOM cultures (Benito Merino et al., 2022). While the identity of these unknowns would be of interest, as they may represent previously undescribed disaccharides, the sheer amount of pure material required for structural determination methods such as NMR precluded structural identification of the unknown metabolites in this study. With determined cultivation efforts and time, enough material may be produced for structural determination. These efforts are further complicated by uncertainty about whether community members produce the disaccharide, so we considered which organism in the enrichment might be responsible.

While direct evidence for the identity of the disaccharide producer in the consortium is lacking, our results suggest that, in the tested cultures, the dominant ANKA, rather than their partner bacteria or community heterotrophs, may produce the main disaccharides. This interpretation is based on comparisons of thermophilic enrichments that share the same partner bacterial species but differ in the dominant sugars in their metabolite profiles (Laso-Pérez et al., 2016; Hahn et al., 2020). In all of these culture, the partner bacteria encode trehalose metabolism, but only in the E50 culture, where the archaeon also has trehalose metabolism, is trehalose the dominant disaccharide. In contrast, in the other thermophilic cultures (GB50, Butane50, and Propane50), trehalose was rare. Here, in the ANKA, HMM hits to trehalose metabolism were also sparse or even absent. However, this study used enrichment cultures, and HMM hits matching those of deposited genomes should not be taken as evidence of *in situ* activity. Therefore, we cannot rule out alternative sources of disaccharide production in these cultures.

In a parallel study, we investigated the presence and production of trehalose in AOM-active marine sediments (Stock et al., 2026). That study found that in AOM-active sediments, the disaccharide production was similar to the fatty acid production of fatty acids produced by the SRB partner, and that the carbon isotopic signatures of disaccharides are more similar to those of SRB-associated lipids, whereas ANME-associated lipids are distinctly more ^13^C-depleted, suggesting that the partner bacteria produce the trehalose. However, this interpretation relies on the assumption that the archaea exhibit identical δ^13^C values in lipids and other metabolites. This is not always the case, as shown for the methanogen *Methanosarcina barkeri,* which, under certain substrate conditions, produces lipids with substantially more negative δ^13^C values than that of bulk biomass (Londry et al., 2008). If phylogenetically related ANMEs exhibit similar isotope fractionation patterns, the *in-situ* isotope data would be consistent with trehalose production by the archaeal partner. The indications of recurrent HGTs between the consortial partners, particularly genes encoding sugar metabolism, make the production of disaccharides by both consortial partners feasible, further complicating the interpretation of the trehalose isotope signals.

### 4.2 Role of disaccharides in alkane-oxidizing cultures

We demonstrated substantial accumulation of trehalose and other disaccharides in enrichments dominated by ANKA, yet the functional roles of the disaccharides in alkane-oxidizing cultures remain unresolved. Here, we speculate on the function of disaccharides in the culture organisms. One potential role of these disaccharides could be as a carbon and energy reserve. In the presence of sulfate, the ANKA could metabolize disaccharides glycolytically, forming acetate, which could then be oxidized to CO_2_, with reducing equivalents transferred to the partner bacterium. In the absence of sulfate, ANKA could potentially ferment the disaccharide and emit compounds such as lactate or acetate. For SRB, disaccharide energy storage is unexpected as SRB typically favor glycogen (Stams et al., 1983) or polyhydroxyalkanoates (PHAs) as storage compounds (Hai et al., 2004). Trehalose is a well-known osmolyte in marine organisms (Gregory and Boyd, 2021; McParland et al., 2021; Poli et al., 2017), so we also considered the possibility of the disaccharides acting as osmolytes. However, our alkane-oxidizing cultures were maintained at a constant salinity close to seawater, closely mimicking the organisms’ native conditions, making osmotic stress unlikely to drive these extreme accumulations of trehalose or yet-unknown disaccharides. Future experiments should test whether AOM biomass releases sugar-derived CO_2_ or fermentation products when starved of electron acceptors or donors, and whether disaccharide concentrations change in response to salinity.

It could also be possible that saccharides serve as precursors for extracellular polymeric substances (EPS). EPS is known to play a role in ANME consortia and has been suggested to participate in microbially induced silica precipitation (Osorio-Rodriguez et al., 2023). While the 1,1-glycosidic linkage of trehalose precludes linear polymerization, certain trehalose-maltose phosphorylases or maltose-forming trehalose isomerases can interconvert trehalose and maltose, facilitating the formation of 1,4-linked polymers (Koh et al., 2003). Trehalose could serve as a basis for other polymers linked by compounds such as quinones. Such polymers have been synthesized by humans (Kukowka and Maślińska-Solich, 2010; Oliveri et al., 2016; Vinciguerra et al., 2022) but have not been reported from natural environments. These hypothetical polymers might be of interest to industry, and their production in ANKA consortia could offer an opportunity to generate valuable products from short-chain alkane waste streams, such as those from natural gas production. A pure carbohydrate-based EPS would have poor electron-transfer capacity (Gao et al., 2019). However, by incorporating electron carriers into their EPS matrix would give this material semiconducting, polymer-like properties (Yang and Antonietti, 2020), which might support direct interspecies electron transfer between the consortial partners. Such an EPS could also explain reports of “amorphous carbon” in ANME and methanogens (Allen et al., 2021): sequential boiling of polysaccharides, especially in the presence of iron-sulfide minerals and quinones could transform the EPS into sulfurized, humic-like, base insoluble residues (Raven et al., 2021; van Dongen et al., 2003), with Raman spectra resembling those used to justify the presence of allotropic carbon in ANME (Sánchez-Cortés et al., 1998; Yang et al., 1994); with the additional caveat that Li et al. (2023) have reported that Raman spectroscopy can induce the D and G bands used to identify carbonaceous compounds in otherwise non-carbonaceous organic matter (Li et al., 2023).

While the function of disaccharides in ANKA/SRB consortia remains speculative, their exudation appears to have substantial effects on the surrounding microbiome. The rapid consumption of exogenously supplied trehalose by community heterotrophs indicates that these organisms are well adapted to sudden availability of disaccharides, likely using trehalose as both a carbon and an energy source. However, in alkane-oxidizing cultures, the disaccharides accumulate, suggesting that the carbohydrates are not readily accessible to heterotrophs (i.e., they are intracellular or present in EPS that is difficult to degrade). The weak but detectable incorporation of labeled trehalose into archaeal lipids in an ethane-oxidizing culture, when no other exogenous energy source was provided, led us to consider the possibility that ethane-oxidizing archaea or their partner SRB may grow glycolytically on exogenously supplied carbohydrates, as suggested for ANME by Yin et al. (2022). However, this incorporation could also be caused by the ethane-oxidizing archaea liberating energy stores in the absence of ethane and fixing small amounts of CO_2_ from a DIC pool labeled by the glycolytic oxidation of exogenously provided labeled trehalose by community heterotrophs. Alternatively, the biphytanes we use as diagnostic for *Ca.* Ethanoperedens could also be shared with low-abundance heterotrophic archaea that also grow in the culture, and may be the source of the detected ^13^C label uptake (Hahn et al., 2020; He et al., 2022).

Deep-sea sediments contain high numbers of heterotrophic microorganisms while being poor in potential substrates (Arnosti et al., 2019). Recent work suggests that some heterotrophs grow on AOM-derived proteinogenic necromass (Zhu et al., 2022). Trehalose and other disaccharides are also readily utilized as carbon sources by diverse heterotrophic populations (Lian et al., 2021). The presence of such heterotrophs in our enrichments (Laso-Pérez et al., 2016; Hahn et al., 2020), which immediately consume the provided trehalose, suggests that metabolites produced by ANKA-SRB consortia may support parts of the surrounding heterotrophic sediment communities.

## 5 Conclusion

The persistence of a large disaccharide pool despite active heterotrophs suggests an active sugar economy sustained by the ANKA–SRB consortia. The ANKA consortia may sustain a deep-sediment “barrier biome” in which the oxidation of alkanes is coupled with the secretion of disaccharides that support a metabolically integrated assemblage of secondary heterotrophs. In this model, distinct ANKA–SRB pairings generate characteristic sugar signatures that shape the surrounding microbial community. What, if anything, the microbial community provides in return is unknown. Although these observations are derived from enrichment cultures, they point to a previously unrecognized *in situ* carbon exchange system. Deciphering this metabolic economy by extending this research from enrichment cultures to *in-situ* conditions will refine our models of sedimentary carbon cycling and reveal how one of Earth’s largest microbial habitats manages its carbon flux.

## Data Availability

The raw data supporting the conclusions of this article will be made available by the authors, without undue reservation.
