# Supplementary material for: Alkane-oxidizing consortia produce substantial amounts of disaccharides
Source: Front Microbiol. 2026 Sep 9;17:1877669. doi: 10.3389/fmicb.2026.1877669 (PMC13597711; doi:10.3389/fmicb.2026.1877669)
Supplement: Supplementary file 1 [file Supplementary_file_1.DOCX]

Supplementary Material

# 1 Supplementary Figures and Tables

Figure S1: Chromatogram and Mass spectra of the Galactopyranosyl-D-mannopyranose (GPMG) standard. Inset on the chromatogram is the Mass spectra corresponding to the peak highlighted by a dashed box.

Figure S2: Sulfide production measured over time in incubations of Ethane50 with or without ethane and with and without trehalose, including a medium blank with trehalose.

Figure S3: Medium DIC concentrations at the start and end of incubations for Ethane50 enrichment cultures incubated with or without ^13^C labelled trehalose.

Figure S4: δ^13^C values for the medium DIC at the start and end of incubations for Ethane50 enrichment cultures incubated with or without ^13^C labelled trehalose.

Figure S5: Sulfide production in Ethane50 enrichment cultures incubated with labelled DIC with or without ethane.

Figure S6: DIC concentrations in medium of Ethane50 cultures incubated with or without labelled DIC and with or without ethane. Note that the DIC+ Et+ sample was lost due to an equipment failure.

Figure S7: δ13C values for the medium DIC at the start and end of incubations for Ethane50 enrichment cultures incubated with or without 13C labelled DIC.
